# Risk assessment and prediction of nosocomial infections based on surveillance data using machine learning methods

**DOI:** 10.1186/s12889-024-19096-3

**Published:** 2024-07-04

**Authors:** Ying Chen, Yonghong Zhang, Shuping Nie, Jie Ning, Qinjin Wang, Hanmei Yuan, Hui Wu, Bin Li, Wenbiao Hu, Chao Wu

**Affiliations:** 1 Department of Laboratory Medicine, The Eighth Affiliated Hospital, Sun Yat-sen University, Shenzhen, 518003 PR China; 2 Department of Medical Affairs, People’s Hospital of Ningxia Hui Autonomous Region, Yinchuan, 750004 PR China; 3 School of Public Health and Social Work, Institute of Health and Biomedical Innovation, Queensland University of Technology, Brisbane, Australia

**Keywords:** Nosocomial infections, Hospital-acquired infections (HAI), Prediction, Machine learning, Early warning

## Abstract

**Background:**

Nosocomial infections with heavy disease burden are becoming a major threat to the health care system around the world. Through long-term, systematic, continuous data collection and analysis, Nosocomial infection surveillance (NIS) systems are constructed in each hospital; while these data are only used as real-time surveillance but fail to realize the prediction and early warning function. Study is to screen effective predictors from the routine NIS data, through integrating the multiple risk factors and Machine learning (ML) methods, and eventually realize the trend prediction and risk threshold of Incidence of Nosocomial infection (INI).

**Methods:**

We selected two representative hospitals in southern and northern China, and collected NIS data from 2014 to 2021. Thirty-nine factors including hospital operation volume, nosocomial infection, antibacterial drug use and outdoor temperature data, etc. Five ML methods were used to fit the INI prediction model respectively, and to evaluate and compare their performance.

**Results:**

Compared with other models, Random Forest showed the best performance (5-fold AUC = 0.983) in both hospitals, followed by Support Vector Machine. Among all the factors, 12 indicators were significantly different between high-risk and low-risk groups for INI (*P* < 0.05). After screening the effective predictors through importance analysis, prediction model of the time trend was successfully constructed (R^2^ = 0.473 and 0.780, BIC = -1.537 and -0.731).

**Conclusions:**

The number of surgeries, antibiotics use density, critical disease rate and unreasonable prescription rate and other key indicators could be fitted to be the threshold predictions of INI and quantitative early warning.

**Supplementary Information:**

The online version contains supplementary material available at 10.1186/s12889-024-19096-3.

## Background

Nosocomial infections, also known as hospital-acquired infections(HAI) are becoming a major threat to the health care system around the world [1, 2]. Due to its great impact on morbidity and mortality, the patients with HAI may have a prolonged hospitalization and poor prognosis [3]. HAI even led to outbreaks of nosocomial infections, causing more disease and economic burden for both patients and the health care system [4]. Thus, consistent effort for the prevention of HAI and decrease of nosocomial infections has been taken by governments and the health care systems. 3.2% of hospitalized patients in 2015 have contact with HAI compared with 4% in 2011 [5]. Despite efforts to control infection in developed countries, HAI still caused around 37 000 deaths in Europe each year [6]. While in China, according to a large multicenter epidemiological survey, 3.60% of hospitalized patients had been exposed with HAI [7].

Previous researches on nosocomial infections have confirmed that there is a link between the development of antimicrobial resistance in pathogenic bacteria and HAI [8, 9]. Other related risk factors like prolonged hospital stay [10], stays in ICU [11], and invasive procedures were associated with HAI as well [12]. Ventilator-associated pneumonia and catheter-associated urinary tract infection, are regarded as the common nosocomial infections [13]. Clearly, the causes of nosocomial infections are complex and diverse. Therefore, to fulfill the purpose of prevention and control of HAI, tons of related risk factors should be considered comprehensively.

Nosocomial infection surveillance (NIS) has been proved to be a positive measure to decrease HAI [14]. Through a long-term, systematic, continuous collection and analysis of the rate and quantity of occurrence or distribution in a specific population, data and reports of NIS are sent to the hospital-related authorities, to provide valid information support for better prevention and control of HAI [6]. What is unsatisfactory is that all these data are currently only used as part of the real-time monitoring system, and it is impossible to achieve prediction or early warning.

Machine learning, as a major component of artificial intelligence, has been applied to health service research. It performs well in identifying new variables, visualizing generation, and exploring linear and nonlinear interactions to improve the accuracy of outcomes [15]; Especially in terms of predicting the patient outcome or diagnosis, such as based on clinical data from Electronic Health Record (EHR), it helps distinguish similar diseases or improve informed decision making before the surgery [16–18]. However, data from the hospital rather than patients themself seems to have been abandoned. Data of NIS due to its complexity, heterogeneity and huge size, which has caused its poor adaption in using other prediction tools. Currently, it is hard to fit into linear or quantitative relationships between NIS data and HAI by traditional statistical methods. Machine learning methods exhibit superior performance in fitting nonlinear relationships and in the selection of variables [19]. However, the comparative analysis of their predictive performance in surveillance data has not been thoroughly conducted in such studies.

This study employs four prevalent ML methods alongside one traditional statistical model to construct predictive models for HAI. The aim is to assess and compare the predictive efficacy and performance of various model types. Additionally, the predictive models discern pertinent risk factors for HAI from the NIS database, thereby optimizing the utilization of NIS data and providing reference significance for the prevention and management of nosocomial infections. Notably, this study incorporates databases from two hospitals simultaneously, allowing for a comparison of the model performance and risk factors across different hospitals to determine whether potential differences exist.

## Methods

### Study setting

We chose two hospitals located in southern and northern China as the study site, that differ greatly in terms of climate and economic levels. One is a large teaching hospital located in Shenzhen, a megacity in southern China (22°38′N, 114°05′E). Shenzhen has the third-largest GDP in China with a permanent resident population of 17.56 million (according to 2020 statistics). This study setting (hereinafter referred to as Hospital 1, H1) is an affiliated hospital of a top comprehensive university located in the central urban area of city. Another is a provincial hospital (hereinafter referred to as Hospital 2, H2), located in northern China (38°47′N, 106°27′E), Yinchuan city. Compare with Shenzhen, Yinchuan city only has a permanent resident population of 0.29 million (according to 2020 statistics) and a much lower GDP. Although the two hospitals differ greatly in their natural and social environments, the number of outpatient and inpatient in these two hospitals were comparable. We hope that they could represent the most hospitals in China as much as possible to improve the representativeness and generalization of the analysis results.

### Data collection

We retrospectively collected monthly information of number and incidence of nosocomial infection from Hospital 1 (January 2014 to April 2020) and Hospital 2 (January 2015 to April 2021), as well as related data with the nosocomial infection surveillance report during the same period, respectively; Specifically including 39 factors (refer Appendices Table S1 for details in the supplemental material), namely hospital operation volume (15 variables, x1–x15), nosocomial infection (8 variables, g1–g8), antibacterial drug use (10 variables, y1–y10) and the number of patients with multidrug-resistant bacteria (c4). Considering the influence of climate, we also collected the outdoor temperature data, including average daily temperature (TAVE), daily maximum temperature (TMAX), and daily minimum temperature (TMIN). These five groups of factors were determined as continuous predictors (independent variables), and the number of patients with nosocomial infection (NNI) and the incidence of nosocomial infections (INI) was set as the prediction target (dependent variable, c1-c3). All variables were organized into a monthly database. We performed the logarithmic transformation using the natural logarithm (base *e*) due to the large differences in the order of magnitude among the factors.

### Statistical analysis

Comparison and evaluation of multiple models.

We used Spearman correlation analysis to explore that these factors were not highly correlated with INI and NNI (Appendices Fig. S1). Logistic regression models and four most common Machine Learning methods were chosen furtherly: Logistic regression (LR), Decision tree (Dtree), Conditional inference tree (Ctree), Random Forest (RF) and Support vector machine (SVM) [17, 20]. We convert INI according to the median (non-normal distribution) or mean (normal distribution) into binary dependent variables (high-risk and low-risk level) as the predicted outcome, and split all data into training set and test set (70%/30% split). Furthermore, the predictive accuracy of five models were assessed using internal cross-validation for two hospitals, respectively. Other results showed that the testing performance can vary depending on the data split. Therefore, it is important to employ multiple data splits when estimating generalization performance [16, 21]. To evaluate the variation in the estimated performance, we calculated the range of AUROC values and reported on the average performance and standard deviation for each model using 3-fold and 5-fold cross-validation.

We trained five different models using the training data and tuned hyperparameters for each model. The hyperparameter value that leads to the best predictive performance was selected by using the performance metric (sensitivity, specificity, positive predictive value, negative predictive value, and accuracy).

Predictors selected by Importance analysis.

Based on above comparisons, the RF model was chosen for its best performance. The RF model’s importance analysis was then used to evaluate the significance of variables (details of the comparative analysis are provided in the Results section). We evaluated the importance of variables by calculating and ranking Increase in mean square-error (%IncMSE) and Increase in node purities (IncNodePurity), which related to the loss function and selected the loss function through the best segmentation. It evaluated multivariate importance by removing predictor variables from each single tree in the forest with the RF model and to measure the change in accuracy to evaluate the effect of the predictor variables. More useful variables achieve higher %IncMSE (Appendices Table S2) [22]. To enhance the stability and accuracy of the RF model and prevent overfitting, we employed several strategies, including: increasing the number of trees (n_estimators) to 500 for better prediction averaging and reduced variance; limiting the number of variables considered at each split (max_features) to 3 to encourage diversity among trees and reduce correlation; and imposing a maximum depth restriction (max_depth) to prevent excessive complexity and overfitting to training data.

Trend prediction and risk threshold prediction.

Autoregressive integrated moving average (ARIMA) is a statistical analysis model that uses time-series data to either better understand the data set or to predict future trends. We used INI of two hospitals as the dependent variable, factors with higher %IncMSE according to Importance ranking of RF analysis as the independent variables. ARIMA makes use of lagged moving averages to smooth time series data. An autoregressive notation (p), a differencing notation (d) and a moving average notation (q) will form the multiplicative process of ARIMA as (p, d, q) [23]. An ARIMA model can be considered as a good model if it has a large stationary R square (R^2^) value and small Bayesian Information Criteria (BIC) and Root Mean Square Error (RMSE).

Classification and regression trees can create a binary tree; each node has exactly two outgoing edges, finding the best categorical or numerical feature to split using an appropriate impurity criterion. The independent variable can be a categorical (Classification tree) or a continuous variable (Regression tree) [24]. In this study, we performed Regression tree analyses to determine the hierarchical threshold between the NNI and important variables. The model evaluated the quantitative relationship among multiple variables and ranked them from greatest to least, according to the degree of impact, and calculated the risk threshold and the estimated number of cases in different situations.

All the above analysis methods were performed separately in H1 and H2 databases, to ensure a diverse representation of predictability of Nosocomial Infections, which is critical for the generalizability of our ML models. We conducted same modeling analyses using data from two different hospitals, aiming to evaluate whether the best ML models identified in this study also perform well in other hospitals, and whether the factors selected by the models are consistent across different hospitals. Overall, the aim was to assess the generalization of the models constructed and the universality of risk factors screened in this study.

R software version 4.0.3 (The R Project for Statistical Computing, Vienna, Austria) was used for the establishment and comparison of models based on Machine Learning. The following R packages were used for these approaches: GGally package for correlation analysis; forecast package for ARIMA analysis; glm package for logistic regression; rpart, rpart.plot packages for decision tree model; party package for conditional inference tree; randomForest package for random forest; and e1071 packages for support vector machine; PROC and ROCR packages for receiver operating characteristic (ROC) curve analysis. SPSS version 25.0 for Windows (SPSS Inc., Chicago, IL, USA) was used for the ARIMA analyses.

## Results

### Comparison and evaluation of multiple models

RF has higher predictive power than the other four methods, as shown in Tables 1 and 2, and Appendices Fig. S2. AUC value of five models are all higher than 0.5, and the RF and SVM are both higher than 0.7. The RF model had the best predictive performance with an AUC value of 0.750 and 0.834 in H1 and H2, respectively (95% confidence interval, CI [95%CI], 0.546–0.954 and 0.621-1.000). The differences between multiple splits were relatively small and gradually increases, indicating that the generalization performance of the model was better. Still, RF was the most accurate prediction model compared to others for H1 and H2 (AUC = 0.938, 0.796-1.000).

**Table 1:** Performance metrics for the best model for each machine learning algorithm

	Sensitivity	Specificity	Positive predictive value	Negative predictive value	Accuracy
**H1**					
Logistic regression	0.58	0.67	0.64	0.62	0.625
Decision tree	0.75	0.58	0.64	0.70	0.667
Conditional inference tree	0.83	0.25	0.53	0.60	0.542
Random forest	0.83	0.67	0.71	0.80	**0.750**
Support vector machine	0.58	0.92	0.88	0.69	**0.750**
**H2**					
Logistic regression	0.4	0.71	0.50	0.63	0.584
Decision tree	0.3	0.79	0.50	0.61	0.583
Conditional inference tree	0.8	0.64	0.62	0.82	0.708
Random forest	0.7	0.93	0.88	0.81	**0.834**
Support vector machine	0.8	0.79	0.73	0.85	0.791

**Table 2:** Comparison Performance Profiles based on cross-validation

	AUC	95%CI	3-fold AUC	3-fold 95%CI	5-fold AUC	5-fold 95%CI
**H1**						
Logistic regression	0.625	0.397–0.853	0.575	0.353–0.797	0.688	0.417–0.958
Decision tree	0.667	0.444–0.889	0.398	0.182–0.615	0.375	0.093–0.657
Conditional inference tree	0.542	0.307–0.777	0.533	0.312–0.754	0.532	0.237–0.826
Random forest	**0.750**	0.546–0.954	0.847	0.682–1.000	**0.938**	0.796–1.000
Support vector machine	**0.750**	0.546–0.954	**0.887**	0.744–1.000	0.875	0.682–1.000
**H2**						
Logistic regression	0.584	0.318–0.796	0.709	0.508–0.909	0.706	0.443–0.969
Decision tree	0.583	0.303–0.783	0.641	0.413–0.869	0.617	0.314–0.919
Conditional inference tree	0.708	0.510–0.933	0.692	0.472–0.911	0.767	0.518–1.000
Random forest	**0.834**	0.621–1.000	**0.926**	0.809–1.000	**0.938**	0.796–1.000
Support vector machine	0.791	0.599–0.986	0.804	0.612–0.995	0.817	0.593–1.000

### Predictors selected by importance analysis and comparative analysis

**Fig. 1:**
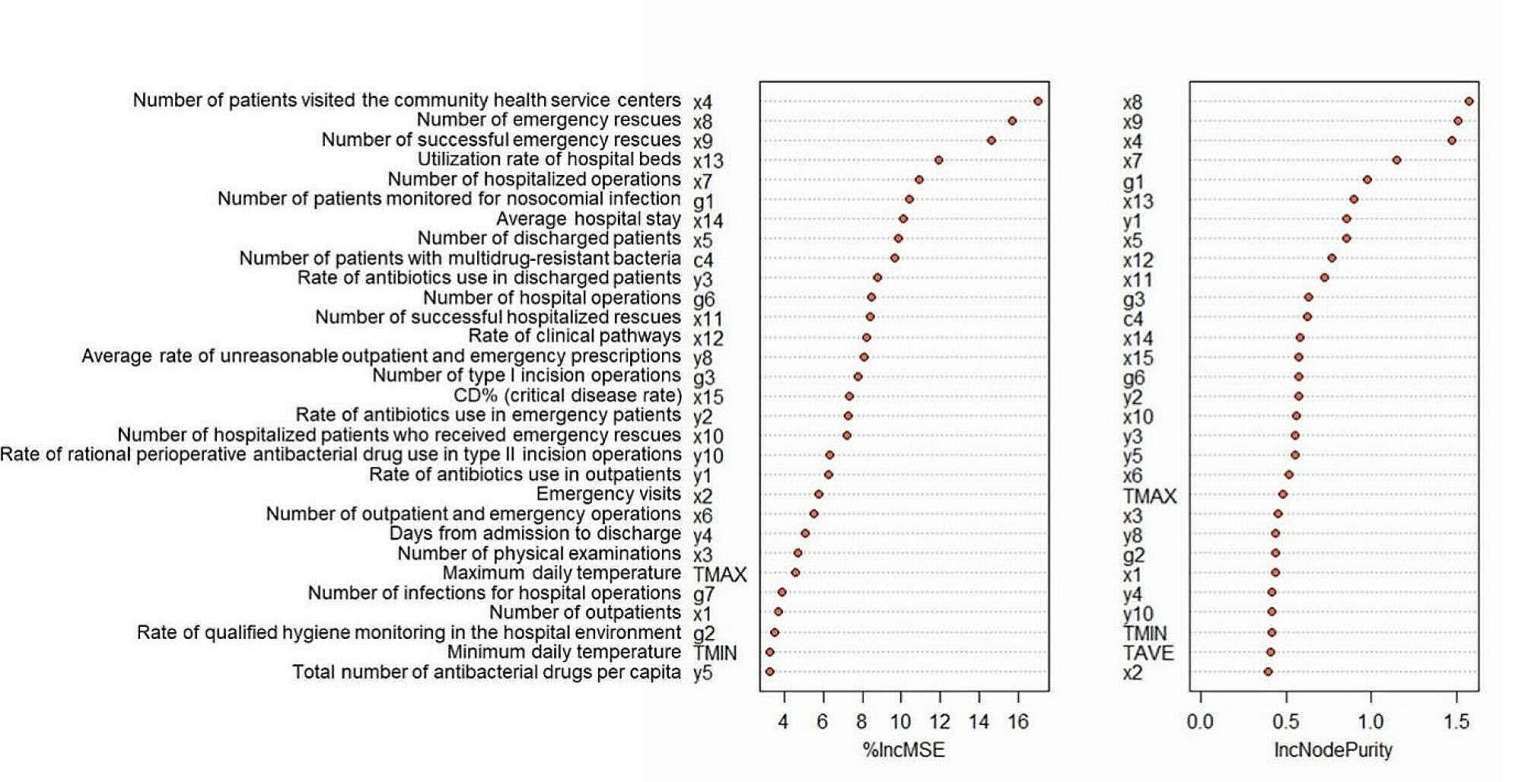
Importance ranking of influencing factors used by the random forest model for predicting the incidence of nosocomial infections (INIs)

We used RF model with the best predictive performance and merged data of H1 and H2 to select the significant influencing factors for nosocomial infection; its results of Importance Ranking (Fig. 1) showed that important factors include indicators mainly related to the number of hospital admissions (e.g., x4, x8, x13, x7, x5), multi-drug resistance (c4) and antibiotic use (y3), etc., which had a higher degree of importance in terms of INI.

**Fig. 2:**
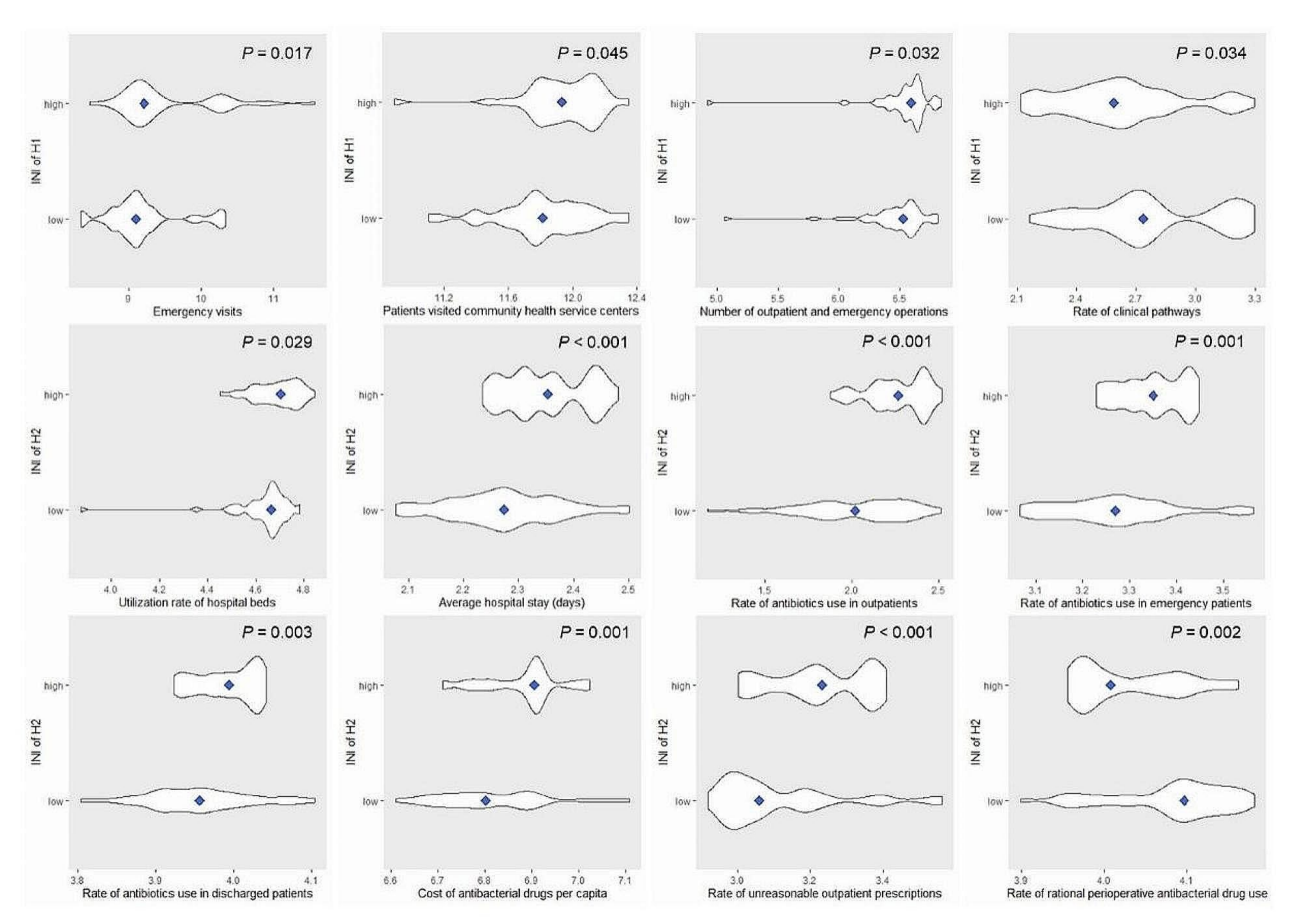
Violin plots show the difference in significant factors between high-risk and low-risk level Blue dots represent the median or mean

The t-test or Mann-Whitney U test analysis showed the significance of the difference between the high-risk and low-risk levels of INI for these 12 factors with *P* < 0.05 (Fig. 2). Emergency visits, Patients visited community health service centers, the Number of outpatient and emergency operations were positively correlated with the INI; and the Rate of clinical pathways were negatively correlated with the INI for H1. For H2, the Utilization rate of hospital beds, Average hospital stay (days), Rate of antibiotics use in outpatients, Rate of antibiotics use in emergency patients, Rate of antibiotics use in discharged patients, Cost of antibacterial drugs per capita, Rate of unreasonable outpatient prescriptions were positively correlated with the INI; and Rate of rational perioperative antibacterial drug use were negatively correlated with the INI.

### Trend prediction and risk threshold prediction

**Fig. 3:**
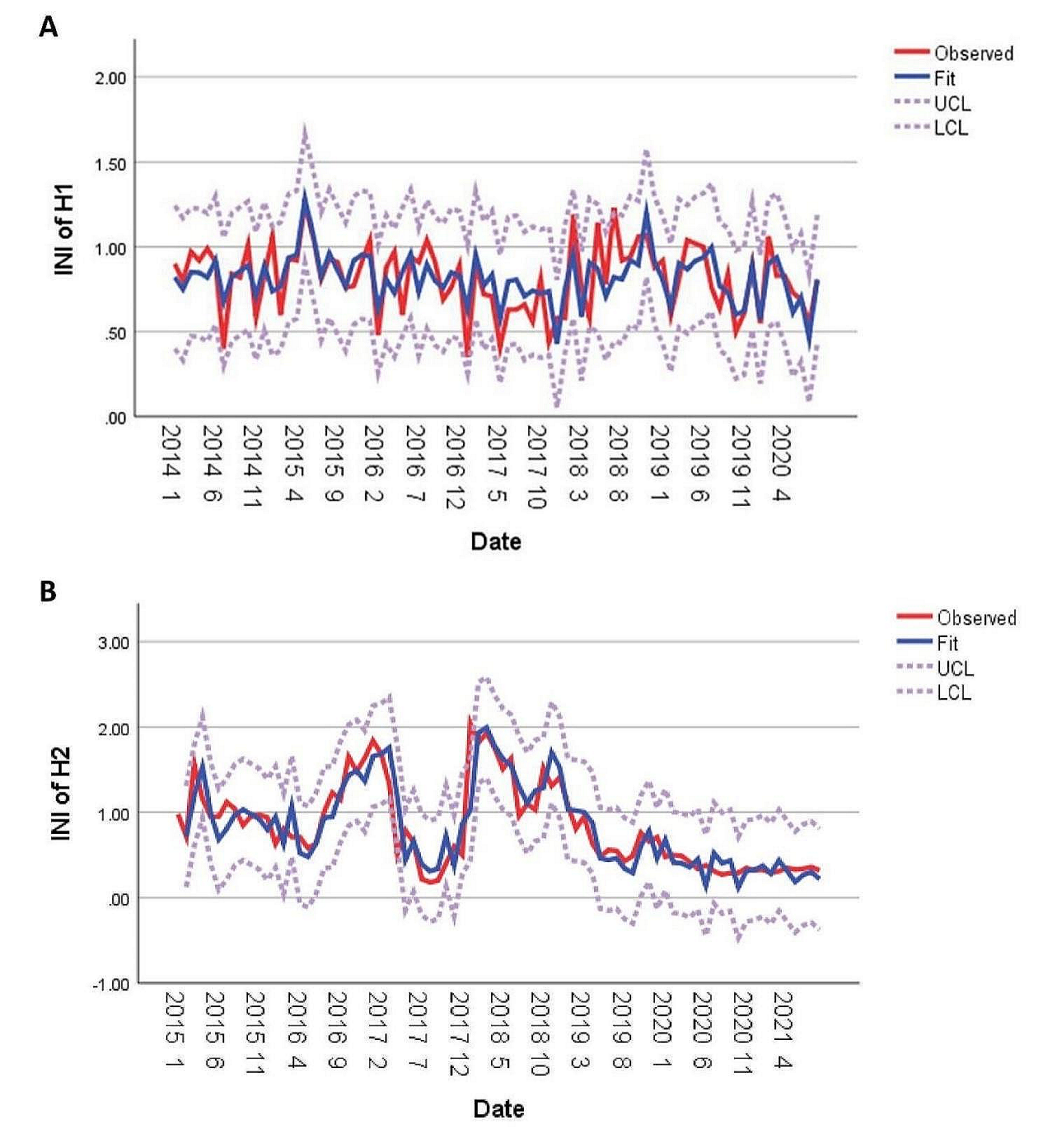
Trend prediction of the number of nosocomial infections established using the autoregressive integrated moving average model (ARIMA)

We used the R software function package to realize the automatic selection of the optimal exponential model for INI of H1 and H2, respectively, and included the top 15 factors of %IncMSE according to Importance ranking of RF showed in Appendices Fig S3 and Table S3 as the predictive variables. Two ARIMA models were built for time series prediction. One was ARIMA (2,0,0) model for H1 with R^2^ = 0.473; another was ARIMA (0,1,0) for H2 with R^2^ = 0.780 (show plots in Fig. 3 and parameters in Table S4). In most years, the actual value of INI is consistent with the predicted value, and a few have slight differences; It indicates that the ARIMA model and variables have a certain reference value for trend prediction.

The actual value of INIs and the fitted diagram of the predicted value of ARIMA in (A) Hospital 1 (2,0,0) and (B) Hospital 2 (0,1,0).

**Fig. 4:**
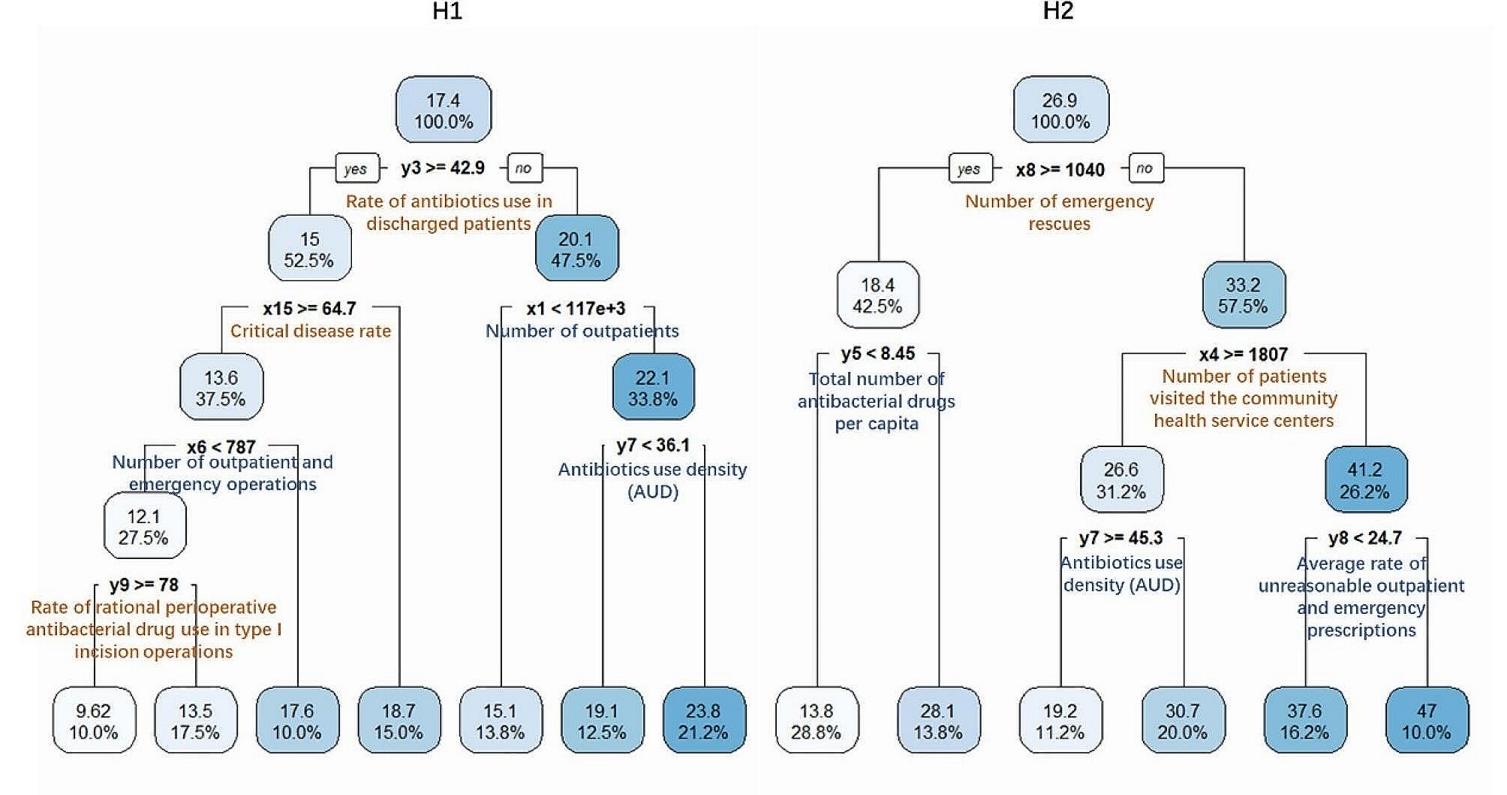
Threshold prediction of the number of nosocomial infections (NNI) established using the regression tree model

A Regression tree auto-selected six factors to build a threshold prediction model for NNI of H1 (Left in Fig. 4), and predicted that the number of people who may have nosocomial infections this month ranges from 6.62 to 23.8 approximately, which judged according to the cut-off range of these six factors. Similarly, five factors were auto-selected to estimate the number of people who may have nosocomial infections in H2 (Right in Fig. 4), ranging from 13.8 to 47 approximately. In Fig. 4, blue factors are positively correlated with NNI, and orange factors are negatively correlated with NNI; those factors can be used to build threshold predictions for NNI of H1 and H2.

Through the threshold of multiple variables, NNI can be divided into high and low levels; blue variables are positively correlated with NNI, and orange variables are negatively correlated with NNI; H1 represents Hospital 1, and H2 represents Hospital 2.

## Discussion

In this study, we present a completely new approach to build a risk assessment system for nosocomial infection based on Machine learning algorithms, which could solve the problems of data fitting and model construction by comparing the performance efficiency among them. Ultimately, we identified the optimal machine learning algorithms for predicting nosocomial infections, along with extrapolating primary risk factors across different hospitals. Random Forest emerged as the most effective model for predicting nosocomial infections with the dimension of incidence rates and case numbers. Factors such as length of hospital stay, antibiotic usage density, multi-drug resistance, hygiene of hospital environmental, number of operations in hospital, rate of unreasonable prescriptions demonstrated significance in predicting the incidence of nosocomial infections.

RF model showed better predictive accuracy and higher AUC for predicting the incidence and the numbers of nosocomial infection than the other models, which indicates the RF model in predicting nosocomial infection has the higher reliability compared with others. RF has shown its potential and superior for predicting the impending occurrence of severe diseases and complications [25]. Such as the postoperative complications [16, 17], or the survival of cancer patients [18], all of which have shown the RF model performs better than the others. For RF selected features by random sampling and random selection and less likely to cause the phenomenon of overfitting, it handles well in anti-noise of nonlinear problems. Plus, it’s dealing well with mixed types of missing data and different forms of predictor variables.

For the relationship between antibiotic use and HAI, the multi-drug resistance problem takes a huge part of it, especially for the ICU patients, who may have a greater chance of being treated with invasive operation and antimicrobials [26]. When the nosocomial infection increases, antibiotic prescribing is tending to be the leading indicator of the hospital for treatment, especially for hospital-acquired pneumonia [27]. The increased number of antibiotic use(y3) in hospitals may base on the empirical antimicrobial therapy, there could be a potential possibility causing the increased inappropriate, excessive or unnecessary number of antibiotic use, which is among the leading causes of the spread of resistance and HAI [28], as the increased number of antibiotic use(y3) has fastened the selection of naturally resistant bacteria that already exists, therefore may be the cause to the increased number of patients with multidrug-resistant bacteria(c4) [29].

The significant factors related to the numbers of hospital admissions, especially average hospital stay (days), have been regarded as the related factors of nosocomial infection. This probably reflected the prolonged possibility of contacting other risk factors for the patients, such as the pathogens. Qualified hygiene monitoring in the hospital environment(g2) also matters as there is evidence like poor hand washing practice among medical, nursing and support staff that may have contributed to the spread of HAI [30, 31]. Increased number of operations in hospital (g6, x7), especially for the type I incision operations(g3), like lobectomy and its related mechanical ventilation, placement of a central venous catheter, as well as the use of a nasogastric tube and urinary tract catheterization, all of which are regarded as responsible for the increased HAI [32]. That’s why the necessary monitoring measures like rate of clinical pathways(x12), qualified hygiene monitoring in the hospital environment matter(g2) and rate of rational perioperative antibacterial drug use in type II incision operations(y10) are needed and are the risk factor of HAI.

Other factors exert influence on the NNI through varied mechanisms, including the frequency of emergency rescues, patient visits to community health service centers, outpatient numbers, and the Critical Disease Rate (CD%). Emergency rescues typically involve critically ill patients who are inherently more susceptible to infections. An escalation in emergency rescues may correlate with heightened NNI, possibly attributable to the swift, high-volume processing of patients, potentially compromising sanitation protocols or curtailing sterilization procedures. Notably, community health service centers refer a considerable volume of patients to hospitals, augmenting patient inflow and, consequently, NNIs. Moreover, elevated outpatient traffic can engender overcrowding, fostering increased inter-patient or patient-healthcare-worker interactions. A heightened CD% may correlate with amplified NNI, given the compromised immune systems of critically ill patients, rendering them more susceptible to infections. Furthermore, prevalent invasive procedures and prolonged hospital stays among critically ill patients further elevate infection risk. Future studies should focus on quantifying the impact of these factors on NNI and devising targeted interventions to enhance patient safety and healthcare outcomes.

For the realization of the risk assessment and trend prediction of nosocomial infection and early warning, data from two representative hospitals in China were collected. Our study indicates that the predictive factors for H1 and H2 are both similar and different, rendering it challenging to achieve successful model construction across different hospitals using the same set of predictive factors. We speculate that this variance may stem from differences in hospital environmental hygiene, differences in clinical specialties, and distribution of disease, among other factors, resulting in varying influences factors on HAIs. While we do not offer a universally applicable predictive index system, we present a series of methodologies for hospitals to establish their own HAI predictive index systems based on their individual characteristics. We highlight the potential benefits of our approach, acknowledging that while this strategy aims to enhance model generalizability, it also presents potential heterogeneity in terms of predictors across different hospital. Cheerfully both their commonalities and unique properties have been screened out. Commonalities can be extrapolated to the other hospitals by using common risk factors for nosocomial infection surveillance and prediction. For their unique indicators, clearly can be regarded as key points for nosocomial infection prevention and early control in each hospital. We have not only done model comparisons and screening of predictive indicators, but also constructed visualization strategies that can be used in practical applications through ARIMA and Regression trees.

Still, the limitations of this study are that it’s based on the ecological approach and the retrospective study of “real world” data; Some influential and more precise factors were not considered comprehensively and partly uncollected, such as the number of visits and antibiotics use in some key departments, like in ICU, etc. Furthermore, there are a limited number of hospitals included in this study. We are expecting that more representative hospitals will be considered in future studies, through which a more widely used risk evaluation system for the early prevention of nosocomial infection can be built up. Future research endeavors are anticipated to undertake prospective studies aimed at assessing the feasibility of utilizing the RF model in conjunction with nosocomial infection risk factors to facilitate early detection and warning of HAI occurrences. Such studies will seek to comprehensively evaluate the predictive accuracy and practical applicability of this integrated approach within clinical settings.

### Electronic supplementary material

Below is the link to the electronic supplementary material.

Supplementary Material 1

## Data Availability

The datasets used and/or analysed during the current study are available from the corresponding author on reasonable request.
