# Supplementary material for: Risk assessment and prediction of nosocomial infections based on surveillance data using machine learning methods
Source: BMC Public Health. 2024 Jul 4;24:1780. doi: 10.1186/s12889-024-19096-3 (PMC11223322; doi:10.1186/s12889-024-19096-3)
Supplement: Supplementary file 1 — Supplementary Material 1 [file 12889_2024_19096_MOESM1_ESM.pdf]

Risk Assessment and Early Warning of Nosocomial Infections Based on Surveillance Data and Machine Learning Methods

Supplementary materials

Table S1

Figure S1

Figure S2

Table S2

Figure S3

Table S3

Table S4

Table S1. List of indicators in the model	
**Variable code**	**Variable description**
**c1**	Number of patients with nosocomial infection
**c2**	Number of nosocomial infection events
**c3**	Incidence of nosocomial infections
**c4**	Number of patients with multidrug-resistant bacteria
**TAVE**	Average daily temperature
**TMAX**	Maximum daily temperature
**TMIN**	Minimum daily temperature
**x1**	Number of outpatients
**x2**	Emergency visits
**x3**	Number of physical examinations
**x4**	Number of patients visited the community health service centers
**x5**	Number of discharged patients
**x6**	Number of outpatient and emergency operations
**x7**	Number of hospitalized operations
**x8**	Number of emergency rescues
**x9**	Number of successful emergency rescues
**x10**	Number of hospitalized patients who received emergency rescues
**x11**	Number of successful hospitalized rescues
**x12**	Rate of clinical pathways
**x13**	Utilization rate of hospital beds
**x14**	Average hospital stay (days)
**x15**	CD% (critical disease rate)
**g1**	Number of patients monitored for nosocomial infection
**g2**	Rate of qualified hygiene monitoring in the hospital environment
**g3**	Number of type I incision operations
**g4**	Number of infections for type I incision operations
**g5**	Rate of infections for type I incision operations
**g6**	Number of hospital operations
**g7**	Number of infections for hospital operations
**g8**	Rate of infections for hospital operations
**y1**	Rate of antibiotics use in outpatients
**y2**	Rate of antibiotics use in emergency patients
**y3**	Rate of antibiotics use in discharged patients
**y4**	Days from admission to discharge
**y5**	Total number of antibacterial drugs per capita
**y6**	Cost of antibacterial drugs per capita
**y7**	Antibiotics use density (AUD)
**y8**	Average rate of unreasonable outpatient and emergency prescriptions
**y9**	Rate of rational perioperative antibacterial drug use in type I incision operations
**y10**	Rate of rational perioperative antibacterial drug use in type II incision operations

A

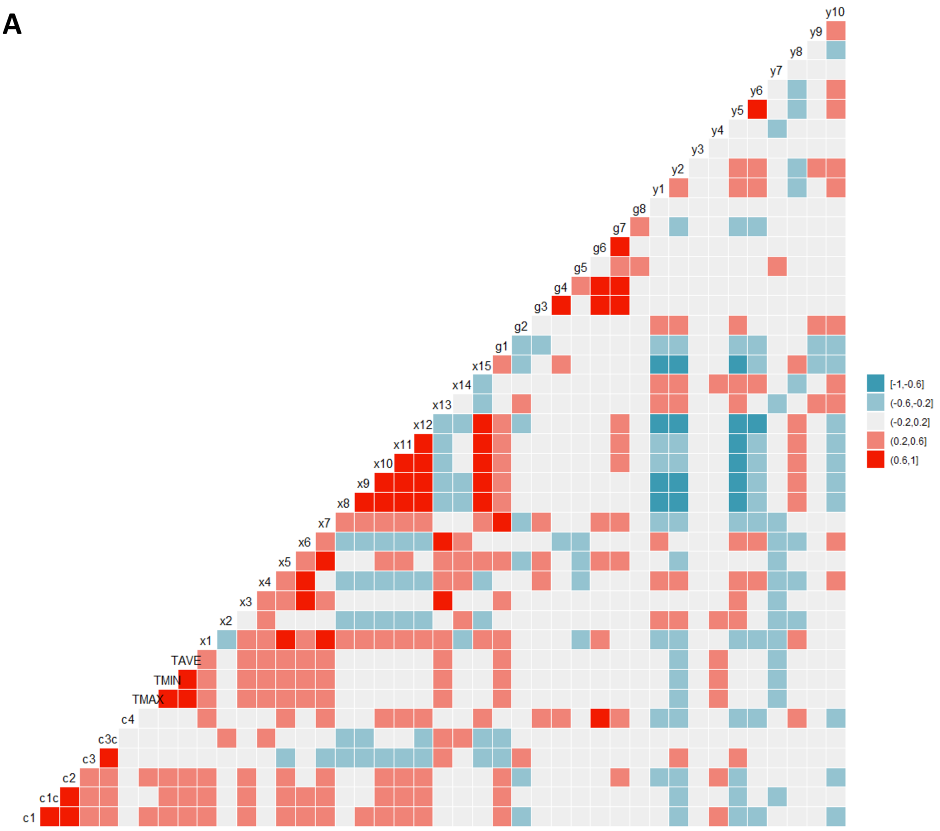

B

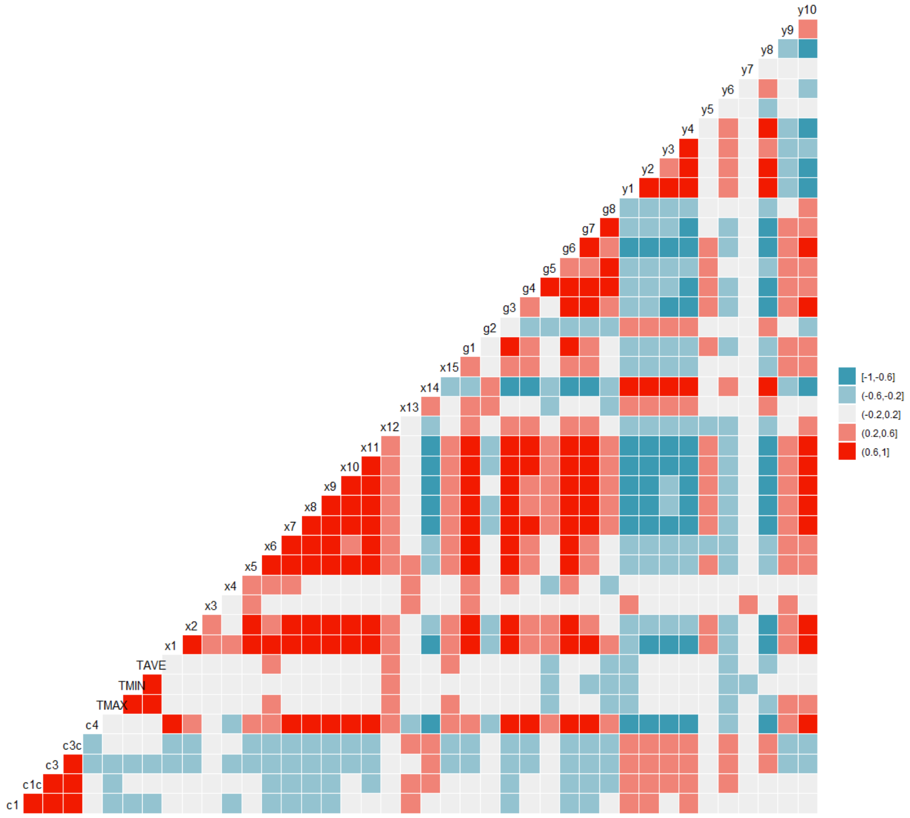

Figure S1. Correlation analysis of all variables for A) Hospital 1 and B) hospital 2

A B

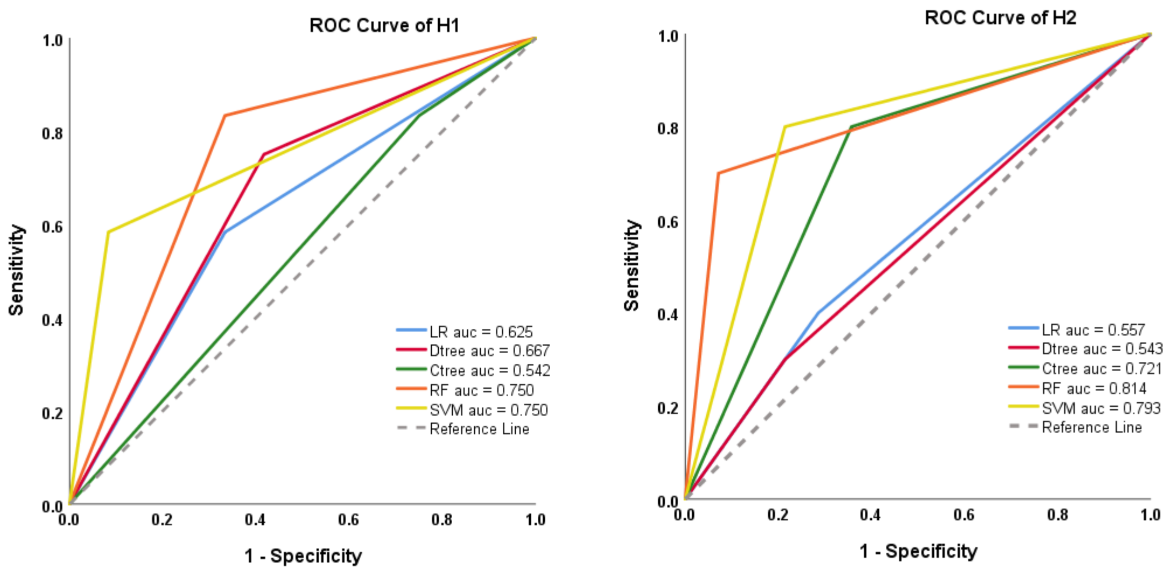

Figure S2. Receiver Operating Characteristic plots for the best machine learning algorithms for A) Hospital 1 and B) hospital 2

Table S2. The coefficient list of importance analysis			
**variable code**	**Variable interpretation**	**%IncMSE**	**IncNodePurity**
c4	Number of patients with multidrug-resistant bacteria	10.24827	0.670224
TMAX	Average daily temperature	3.084049	0.46114
TMIN	Maximum daily temperature	4.364611	0.413556
TAVE	Minimum daily temperature	3.462728	0.43291
x1	Number of outpatients	3.775895	0.398906
x2	Emergency visits	5.307115	0.346887
x3	Number of physical examinations	5.420781	0.422624
x4	Number of patients visited the community health service centers	17.00415	1.401264
x5	Number of discharged patients	10.85833	0.875937
x6	Number of outpatient and emergency operations	5.334705	0.456226
x7	Number of hospitalized operations	11.48561	1.271054
x8	Number of emergency rescues	16.0681	1.528875
x9	Number of successful emergency rescues	15.95194	1.420606
x10	Number of hospitalized patients who received emergency rescues	6.196213	0.558269
x11	Number of successful hospitalized rescues	8.124076	0.758881
x12	Rate of clinical pathways	7.785912	0.877607
x13	Utilization rate of hospital beds	11.54964	0.919855
x14	Average hospital stay (days)	10.62553	0.572463
x15	CD% (critical disease rate)	7.789502	0.598571
g1	Number of patients monitored for nosocomial infection	9.958762	0.999814
g2	Rate of qualified hygiene monitoring in the hospital environment	3.751761	0.500891
g3	Number of type I incision operations	7.070768	0.622073
g4	Number of infections for type I incision operations	2.952428	0.101843
g5	Rate of infections for type I incision operations	2.453139	0.2411
g6	Number of hospital operations	8.063776	0.598106
g7	Number of infections for hospital operations	5.400211	0.185864
g8	Rate of infections for hospital operations	1.727534	0.311066
y1	Rate of antibiotics use in outpatients	6.399039	0.699409
y2	Rate of antibiotics use in emergency patients	8.087283	0.624371
y3	Rate of antibiotics use in discharged patients	9.212643	0.618482
y4	Days from admission to discharge	5.625109	0.420993
y5	Total number of antibacterial drugs per capita	1.74758	0.517106
y6	Cost of antibacterial drugs per capita	2.81942	0.235647
y7	Antibiotics use density (AUD)	0.933199	0.324132
y8	Average rate of unreasonable outpatient and emergency prescriptions	7.953892	0.419619
y9	Rate of rational perioperative antibacterial drug use in type I incision operations	3.162614	0.303599
y10	Rate of rational perioperative antibacterial drug use in type II incision operations	6.009037	0.417598

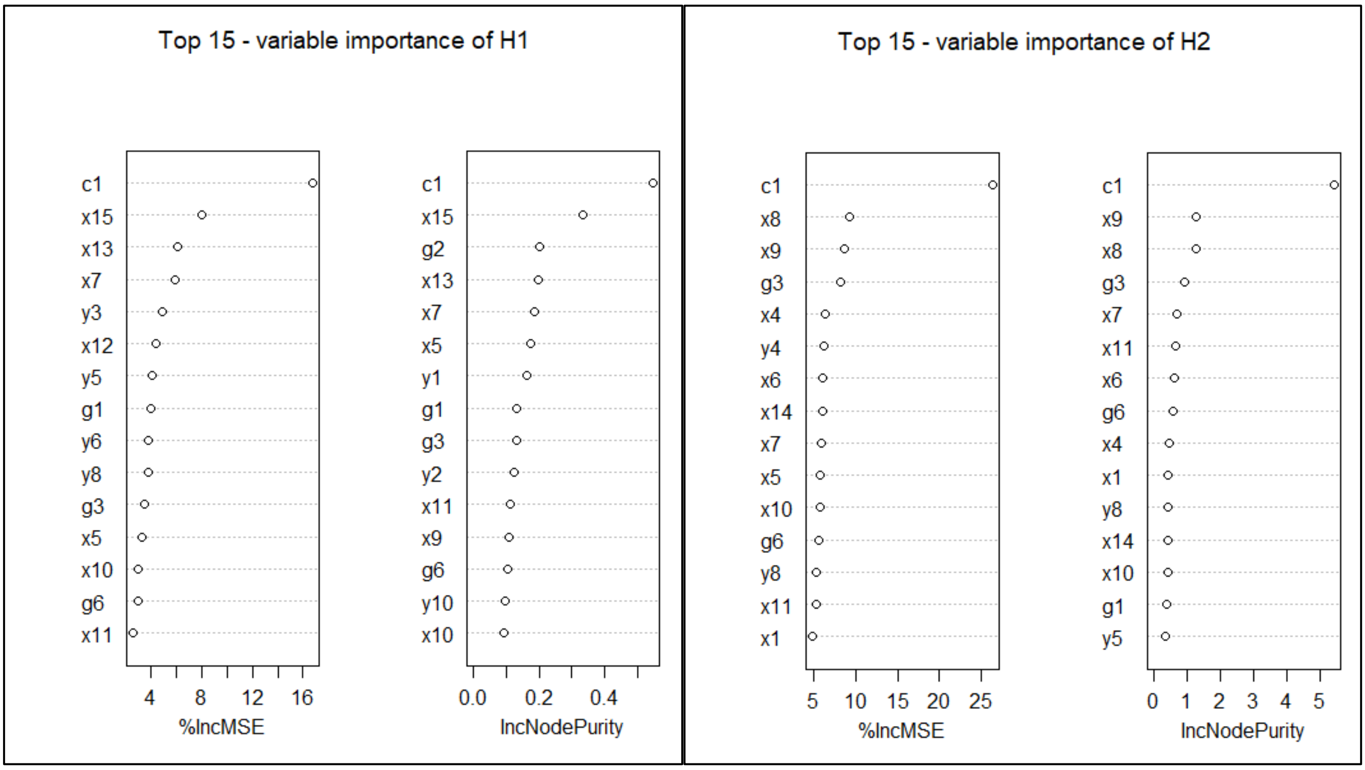

Figure S3. Top 15 influencing factors importance ranking by the random forest model for predicting nosocomial infections (INIs) at hospital 1 and hospital 2

Table S3. Coefficient list of top 15 variable importance of H1 and H2

	variable code	Variable interpretation	%IncMSE	IncNodePurity
H1	c1	Number of patients with nosocomial infection	16.734343	0.545614
	x15	CD% (critical disease rate)	8.039437	0.333790
	x13	Utilization rate of hospital beds	6.116070	0.198620
	x7	Number of hospitalized operations	5.836231	0.186821
	y3	Rate of antibiotics use in discharged patients	4.877753	0.088602
	x12	Rate of clinical pathways	4.355293	0.090565
	y5	Total number of antibacterial drugs per capita	4.023234	0.081295
	g1	Number of patients monitored for nosocomial infection	3.967687	0.131504
	y6	Cost of antibacterial drugs per capita	3.788909	0.076618
	y8	Average rate of unreasonable outpatient and emergency prescriptions	3.720592	0.075607
	g3	Number of type I incision operations	3.414663	0.130103
	x5	Number of discharged patients	3.310349	0.174328
	x10	Number of hospitalized patients who received emergency rescues	2.985450	0.092907
	g6	Number of hospital operations	2.971790	0.104354
	x11	Number of successful hospitalized rescues	2.567760	0.112928
H2	c1	Number of patients with nosocomial infection	26.312831	5.403653
	x8	Number of emergency rescues	9.218073	1.254110
	x9	Number of successful emergency rescues	8.588901	1.255269
	g3	Number of type I incision operations	8.223123	0.917633
	x4	Number of patients visited the community health service centers	6.289541	0.457484
	y4	Days from admission to discharge	6.284728	0.280189
	x6	Number of outpatient and emergency operations	6.124476	0.610959
	x14	Average hospital stay (days)	6.104037	0.406010
	x7	Number of hospitalized operations	5.909153	0.674457
	x5	Number of discharged patients	5.797889	0.322661
	x10	Number of hospitalized patients who received emergency rescues	5.756234	0.402600
	g6	Number of hospital operations	5.528749	0.575505
	y8	Average rate of unreasonable outpatient and emergency prescriptions	5.329622	0.415486
	x11	Number of successful hospitalized rescues	5.238039	0.651766
	x1	Number of outpatients	4.856602	0.418140

Table S4. Parameters of ARIMA models of the incidence of nosocomial infections in two hospitals

	R^2	RMSE	BIC
**Hospital 1**	0.473	0.188	-1.537
**Hospital 2**	0.780	0.294	-0.731
